# Effect of short-term exposure to ambient air pollutants on non-accidental mortality in emergency department visits: a time-series study

**DOI:** 10.3389/fpubh.2023.1208514

**Published:** 2023-06-30

**Authors:** Siting Wang, Yongming Zhang, Xia Li, Jinhua Zhao, Naijian Zhang, Yuming Guo, Jiageng Chen, Yuanyuan Liu, Zhuang Cui, Yuanjun Lyu, Jing Gao, Changping Li, Wenyi Zhang, Jun Ma

**Affiliations:** ^1^Department of Epidemiology and Biostatistics, School of Public Health, Tianjin Medical University, Tianjin, China; ^2^Department of Pulmonary and Critical Care Medicine, Center of Respiratory Medicine, China-Japan Friendship Hospital, National Clinical Research Center for Respiratory Diseases, Beijing, China; ^3^Clinical Pharmacology Department, Zhejiang Hisun Pharmaceutical Co., Ltd., Taizhou, Zhejiang, China; ^4^Department of Epidemiology and Preventive Medicine, School of Public Health and Preventive Medicine, Monash University, Melbourne, VIC, Australia; ^5^Department of Endocrinology, Tianjin Hospital, Tianjin, China; ^6^Thoracic Clinical College, Tianjin Medical University, Tianjin, China; ^7^Cardiovascular Institute, Tianjin Chest Hospital, Tianjin, China; ^8^Chinese PLA Center for Disease Control and Prevention, Beijing, China

**Keywords:** non-accidental mortality, air pollution, short-term exposure, gaseous pollutant, time-series study

## Abstract

**Objectives:**

Exposure to air pollution has been linked to an increased risk of premature mortality. However, the acute effects of air pollution on the risk of non-accidental mortality have not been extensively researched in developing countries, and the findings thus far have been inconsistent. Therefore, this study aimed to examine the association between short-term exposure to six pollutants (PM_2.5_, PM_10_, SO_2_, NO_2_, O_3_, and CO) and non-accidental mortality in Beijing, China.

**Methods:**

Daily data on non-accidental deaths were gathered from 1 January 2017 to 31 December 2018. Air pollution data for the same period were collected from 35 fixed-site air quality monitoring stations in Beijing. Generalized additive models (GAM) based on Poisson regression were used to investigate the association between non-accidental mortality in emergency department visits and the daily average levels of air pollutants.

**Results:**

There were 8,676 non-accidental deaths recorded during 2017–2018. After sensitivity analysis, short-term exposure to air pollutants, particularly gaseous pollutants, was linked to non-accidental mortality. Specifically, for every 10 μg/m^3^ increase (5 μg/m^3^ in SO_2_, 0.5 mg/m^3^ in CO) of SO_2_ (lag 04), NO_2_ (lag 04), O_3_ (lag 05), and CO (lag 04), the relative risk (RR) values were 1.054 (95% CI: 1.009, 1.100), 1.038 (95% CI: 1.013, 1.063), 1.032 (95% CI: 1.011, 1.054), and 1.034 (95% CI: 1.004, 1.066), respectively. In terms of causes of death, short-term exposure to NO_2_, SO_2_, and O_3_ increased the risk of circulatory mortality. Further stratified analysis revealed that the stronger associations were presented in females for O_3_ while in males for CO. People aged 65 and over were strongly associated with ambient air pollution.

**Conclusions:**

Our study showed that ambient air pollutants were associated with non-accidental mortality. Our findings suggested that efforts to control gaseous pollution should be stepped up, and vulnerable groups should be the focus of health protection education.

## 1. Introduction

With urbanization and modernization, China has become one of the countries suffering from the most serious air pollution (1, 2). Air pollution has become the fourth leading cause of mortality in China, and it has been related to an increased chance of premature death due to heart disease, stroke, and lung cancer (3–5). The severity of air pollution in China is evident from the frequent occurrence of haze events in recent years, which has raised significant concern (6). Since the beginning of 2013, the incidence and extent of haze have quickly grown with 75% of cities and 8 million people suffering from haze pollution in China, which presents a significant challenge to public health (7).

In response, China has implemented policies such as the Air Pollution Prevention and Control Action Plan (APPCAP) to enhance air quality and lessen the harmful impacts of pollution on human health (8). However, the air quality in Beijing remains critical, with average pollutant concentrations exceeding the World Health Organization's (WHO) targets (9). Recently, a growing number of epidemiological studies have been done in China to gain a comprehensive understanding of the short-term impacts of air pollution on non-accidental mortality (10, 11). Air pollution in China is complexly characterized by high levels of PM_2.5_ and O_3_ (12). A recent meta-analysis in China confirmed that short-term exposure to O_3_ was associated with an elevated risk of cardiovascular mortality (13). Significant links were found between short-term coarse particulate matter exposure and daily non-accidental mortality in a nationwide analysis of 272 Chinese cities (14). However, the findings on gaseous pollutants (SO_2_ and NO_2_) were not entirely consistent across several studies. For instance, the time-series study conducted in Hefei City showed SO_2_ was linked with non-accidental mortality (15), while studies in Northern China and the Pearl River Delta region reported no association between SO_2_ and mortality (16, 17). Furthermore, the studies from Hong Kong and Taipei City reported a significant increase in respiratory disease mortality associated with short-term NO_2_ exposure (18, 19), while another study conducted in Beijing reported a positive but non-significant association (20). It is noteworthy that very few studies have analyzed directly the association between non-accidental deaths and short-term exposure to air pollutants, where both particulate matter and gaseous pollutants are involved (21, 22). Therefore, it is necessary to conduct more research to better understand the relationship between air pollution and non-accidental mortality in China.

Previous studies have predominantly relied on hospitalization data as the primary data source to investigate the association between air pollution and non-accidental mortality, with limited utilization of emergency department data (23, 24). The study has the advantage of providing the evidence of the association between air pollutants (particulate matter and gaseous pollutants) and emergency non-accidental mortality in Beijing. First of all, emergency data provides faster feedback because emergency services typically respond immediately and record relevant data. This allows us to observe acute health events and mortality cases related to air pollution more promptly. Secondly, since emergency services are accessible to the public and people are more likely to seek emergency assistance during acute conditions, these data can cover a broader range of age groups and populations. Some studies have shown that it is significant to examine emergency department data specifically, as it represents more immediate, acute impacts and may identify vulnerability in specific populations (25, 26). Finally, emergency department data can avoid the interference of cross-regional visits when compared with hospitalization data, and brings the advantage of exposure assessment (27).

As the capital of China, Beijing is situated in northern China, with high population density, high levels of industrialization, long heating hours, and high air pollution levels (28). Therefore, it becomes very important to develop related preventive strategies to protect public health, especially sensitive populations, by estimating the acute health impacts of air pollution. This study performed a time-series analysis to investigate the short-term effects of six air pollutants (PM_2.5_, PM_10_, SO_2_, NO_2_, O_3_, and CO) on non-accidental mortality in Beijing and to explore the populations vulnerable to adverse effects of pollutant exposure.

## 2. Materials and methods

### 2.1. Data collection

The death data during 2017–2018 including the gender, age, and causes of death were collected from Beijing Red Cross Emergency Medical Center, which excluded accidental deaths. Causes of death were classified according to the International Classification of Diseases (ICD-10; non-accidental death: A00-R99, neoplasms disease death: C00–D48, respiratory system disease death: J00-J98, circulatory system disease death: I00–I99). Then, we stratified the non-accidental deaths by sex (male and female), age (< 65 and ≥65 years). This study was approved by the Ethics Committees of Tianjin Medical University (No. TMUhMEC 2021009).

The Chinese National Environmental Monitoring Center provided air pollution data during 2017–2018 through their online platform. For all measurements, they were made in accordance with China's National Air Quality Control standards (GB3095-2012). The 24-h average daily concentrations were calculated from the average of 35 fixed-site air quality–monitoring stations, with an average of one monitoring station per 468.9 km^2^. The 24-h average concentrations of six air pollutants including PM_2.5_, PM_10_, CO, SO_2_, NO_2_, and O_3_ were used for further analysis. For adjustment in the analysis, we also extracted the daily average temperature and relative humidity from the China Meteorological Science Data Sharing Service System (http://data.cma.cn/) between 1 January 2017 and 31 December 2018.

### 2.2. Statistical analysis

A time-series design was used in the research to investigate the relationship between short-term exposure to air pollutants and non-accidental mortality in emergency department visits. Descriptive analysis was used for daily non-accidental deaths, air pollutants (PM_2.5_, PM_10_, SO_2_, NO_2_, O_3_, and CO), and meteorological variables (daily average temperature and relative humidity). We investigated the collinearity between air pollutants and meteorological variables using Spearman correlation analysis. Non-accidental mortality in emergency department visits is a low-chance occurrence in this research, with the distribution roughly following the Poisson distribution. Thus, the assessment of the relationship between the daily non-accidental deaths in the emergency department visits and the daily average concentrations of air pollutants was performed by generalized additive models (GAM) based on Poisson regression. The formulation of this study is as follows:


(1) $$\begin{array}{l} \text{Log }[E(Yt)]=\alpha +\beta Zt+\text{ ns}(\text{Time},df_{1}\text{= 2}\times \text{12})\\ \;+\text{ns}(\text{Temperature,}df_{2}=6)\\ \text{ + ns}(\text{Relative humidity},df_{3}=3)+\text{DOW} \end{array}$$

*E*(*Yt*) represents the expected number of non-accidental deaths in emergency department visits at day *t*; α stands for the model intercept and β indicates the log-relative risk (RR) of daily non-accidental mortality associated with a unit increase of pollutant concentrations (*Zt*); ns shows the natural cubic spline smooth function and *df* is its degree of freedom. The ns (time, *df*_1_) is utilized to consider seasonality and long-term trends. The 12 degrees of freedom per year for the time variable were chosen based on the Akaike information criterion (AIC) minimization and previous research (29). Daily average temperature and relative humidity were included as covariates in the formula. By referring to previous literature (30–32), we chose 6 *df* for daily average temperature and 3 *df* for daily average humidity to account for the possible non-linear confounding effects of meteorological variables. The day of the week (DOW) is controlled as a binary variable.

We separately incorporated the air pollutants into the GAM for exploring the relationship between air pollution and daily non-accidental mortality. To explore the potentially delayed effects, we used single lag exposure (lag 0-lag 5) and cumulative lag exposure (lag 01-lag 05). The concentration of pollutant on that day was considered as lag 0, and the concentration of pollutant on the previous 5th day was considered as lag 5. The 6-day moving average pollutant concentration for the current and prior 5 days was recorded as lag 05. We also conducted stratified analyses by age and sex. The *Z*-test was utilized to compare the differences in groups with the following formula:


(2) $$\begin{array}{l} Z=\frac{\beta_{1}-\beta_{2}}{\sqrt{SE_{1}^{2}+SE_{2}^{2}}} \end{array}$$

As an example of sex-stratified analysis, β_1_ and β_2_ are the effect estimates for males and females, while *SE*_1_ and *SE*_2_ are standard errors.

Sensitivity analyses were performed in this study to ensure the stability of the results. Firstly, we used alternative *df* to control the time trend (10–14), temperature (2–7), and relative humidity (2–7) in single-pollutant models. Secondly, two-pollutant models and multi-pollutant models were used to check the confounding effect on non-accidental mortality from co-pollutants. The pollutants with Spearman correlation values >0.60 were not included in models simultaneously to prevent multicollinearity (33). Since temperature has been found to have prolonged health effects in previous studies (34, 35), we also controlled the potential lagged effects of temperature (up to 28 days). Finally, exposure-response (E-R) curves between non-accidental mortality and six air pollutants were plotted based on GAM.

The results were presented as the estimated RR with the corresponding 95% confidence interval (CI) of non-accidental mortality in emergency department visits for every 10 μg/m^3^ (PM_2.5_, PM_10_, NO_2_, and O_3_), 5 μg/m^3^ (SO_2_) or 0.5 mg/m^3^ (CO) increase in ambient air pollutants. In this study, statistical tests were using two-tailed tests, and *P*-values < 0.05 were considered to be statistically significant. R (version 4.1.3) “mgcv” and “splines” packages (R Development Core Team, Vienna, Austria) were used for statistical analysis.

## 3. Results

The total number of non-accidental deaths was 8,676 during 2017–2018, of which 58.37% were males, 77.11% were older than 65 years of age, and 66.83% died from circulatory diseases (Supplementary Table 1). As shown in Table 1, the 24-h average concentrations were 52.58 μg/m^3^ for PM_2.5_, 80.92 μg/m^3^ for PM_10_, 5.95 μg/m^3^ for SO_2_, 40.63 μg/m^3^ for NO_2_, 0.87 mg/m^3^ for CO, and 61.60 μg/m^3^ for O_3_.

**Table 1:** The summary of descriptive statistics during 2017–2018.

	**Mean**	**SD**	**Min**	**P25**	**Median**	**P75**	**Max**
Total (A00-R99)	11.88	4.41	3.00	9.00	11.00	14.00	32.00
Neoplasms (C00-D48)	0.90	0.94	0.00	0.00	1.00	1.00	5.00
Respiratory system (J00-J99)	0.98	0.99	0.00	0.00	1.00	1.00	6.00
Circulatory system (I00-I99)	7.94	3.54	0.00	5.00	8.00	10.00	24.00
**Air pollutant concentrations (24-h average)**							
PM_2.5_ (μg/m^3^)	52.58	49.00	3.00	20.00	40.00	68.00	430.00
PM_10_ (μg/m^3^)	80.92	68.70	0.00	41.00	65.50	100.00	858.00
SO_2_ (μg/m^3^)	5.95	6.45	1.00	2.00	4.00	7.00	81.00
NO_2_ (μg/m^3^)	40.63	19.58	6.00	27.00	36.00	50.00	145.00
O_3_ (μg/m^3^)	61.60	37.77	3.00	33.00	55.00	84.25	181.00
CO (mg/m^3^)	0.87	0.63	0.20	0.50	0.76	1.02	7.28
**Meteorological measure (24-h average)**							
Daily average temperature (°C)	12.07	11.81	−12.00	0.47	13.72	23.00	30.90
Relative humidity (%)	52.76	19.47	14.67	36.25	50.67	70.08	94.67

**Figure 1:**
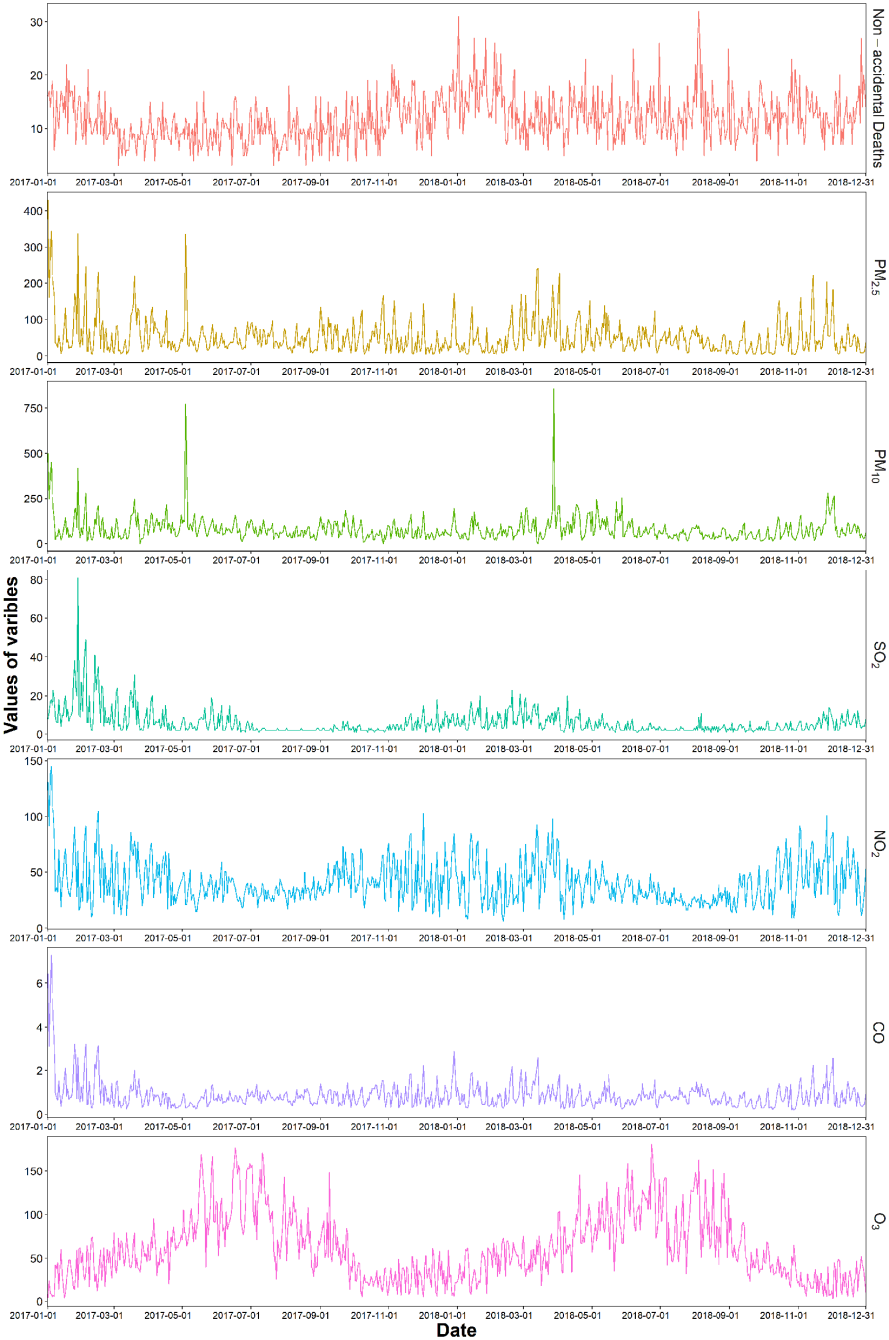
Time series of air pollutants and non-accidental mortality in emergency department visits from 2017 to 2018.

The Spearman correlation analysis results showed there were strong correlations among the air pollutants (Supplementary Table 2). It is worth noting that PM_2.5_ was highly correlated with CO (*r* = 0.85), and PM_10_ (*r* = 0.78). O_3_ was negatively correlated with SO_2_, NO_2_, and CO, respectively (*P* < 0.01). The overall trend in non-accidental deaths from emergency department visits was *U*-shaped, and higher in winter (Figure 1). SO_2_, NO_2_, and O_3_ showed seasonal fluctuations over time with a stable overall trend.

The estimated RRs with 95% CIs for non-accidental mortality in single-pollutant models linked to a unit increment of air pollutant concentrations are shown in Table 2. After adjusting for time-trended, daily average temperature, relative humidity, and day of week, there were significant positive relationships between air pollution and non-accidental mortality. Significant associations between exposure to air pollutants (PM_2.5_, SO_2_, NO_2_, and CO) and non-accidental mortality for emergency department visits could be observed at lag 3 days (lag 4 and lag 5 for O_3_) in single-day lagged models. In contrast to single-day lags, larger effect estimates were observed in multi-day lags. The strongest effects observed for a unit increase in short-term exposure were 1.008 (95% CI: 1.001, 1.016) for PM_2.5_ at lag 04, 1.007 (95% CI: 1.001, 1.013) for PM_10_ at lag 05, 1.054 (95% CI: 1.009, 1.100) for SO_2_ at lag 04, 1.038 (95% CI: 1.013, 1.063) for NO_2_ at lag 04, 1.032 (95% CI: 1.011, 1.054) for O_3_ at lag 05, 1.034 (95% CI: 1.004, 1.066) for CO at lag 04.

**Table 2:** The estimated RRs with 95% CIs of non-accidental mortality in emergency department visits per 10 μg/m^3^ increase in air pollutants (5 μg/m^3^ in SO_2_, 0.5 mg/m^3^ in CO) in single-pollutant models at different lag days.

**Lag days**	**PM_2.5_**	**PM_10_**	**SO_2_**	**NO_2_**	**O_3_**	**CO**
Lag 0	1.004 (0.997, 1.010)	1.003 (0.999, 1.006)	1.006 (0.983, 1.030)	1.010 (0.994, 1.026)	1.006 (0.993, 1.019)	1.019 (0.994, 1.045)
Lag 1	1.001 (0.996, 1.007)	1.003 (0.999, 1.006)	1.009 (0.987, 1.033)	1.006 (0.991, 1.021)	1.008 (0.997, 1.019)	1.011 (0.989, 1.034)
Lag 2	1.003 (0.998, 1.008)	1.003 (1.000, 1.006)	1.013 (0.992, 1.034)	1.008 (0.995, 1.021)	1.007 (0.996, 1.017)	1.014 (0.993, 1.035)
Lag 3	1.006 (1.001, 1.010)^a^	1.003 (0.999, 1.006)	1.028 (1.007, 1.050)^b^	1.022 (1.010,1.035)^b^	1.003 (0.993, 1.014)	1.026 (1.005, 1.047)^a^
Lag 4	1.003 (0.999, 1.008)	1.000 (0.997, 1.004)	1.005 (0.984, 1.027)	1.010 (0.997, 1.022)	1.013 (1.003, 1.023)^a^	1.016 (0.996, 1.037)
Lag 5	1.001 (0.996, 1.006)	1.002 (0.999, 1.006)	0.996 (0.975, 1.018)	1.004 (0.992, 1.017)	1.013 (1.003, 1.023)^b^	1.000 (0.979, 1.020)
Lag 01	1.004 (0.997, 1.010)	1.004 (1.000, 1.008)	1.014 (0.983, 1.046)	1.013 (0.994, 1.033)	1.011 (0.996, 1.026)	1.021 (0.993, 1.049)
Lag 02	1.005 (0.998, 1.012)	1.005 (1.000, 1.010)^a^	1.026 (0.990, 1.064)	1.018 (0.996, 1.040)	1.015 (0.998, 1.031)	1.023 (0.994, 1.053)
Lag 03	1.007 (1.000, 1.015)	1.006 (1.001, 1.012)^a^	1.050 (1.009, 1.092)^a^	1.033 (1.010, 1.057)^b^	1.016 (0.998, 1.034)	1.032 (1.002, 1.064)^a^
Lag 04	1.008 (1.001, 1.016)^a^	1.006 (1.000, 1.012)^a^	1.054 (1.009, 1.100)^a^	1.038 (1.013, 1.063)^b^	1.025 (1.005, 1.045)^a^	1.034 (1.004, 1.066)^a^
Lag 05	1.008 (1.000, 1.017)	1.007 (1.001, 1.013)^a^	1.052 (1.003, 1.103)^a^	1.037 (1.012, 1.064)^b^	1.032 (1.011, 1.054)^b^	1.032 (1.000, 1.064)^a^

^a^*p* < 0.05.

^b^*p* < 0.01.

**Figure 2:**
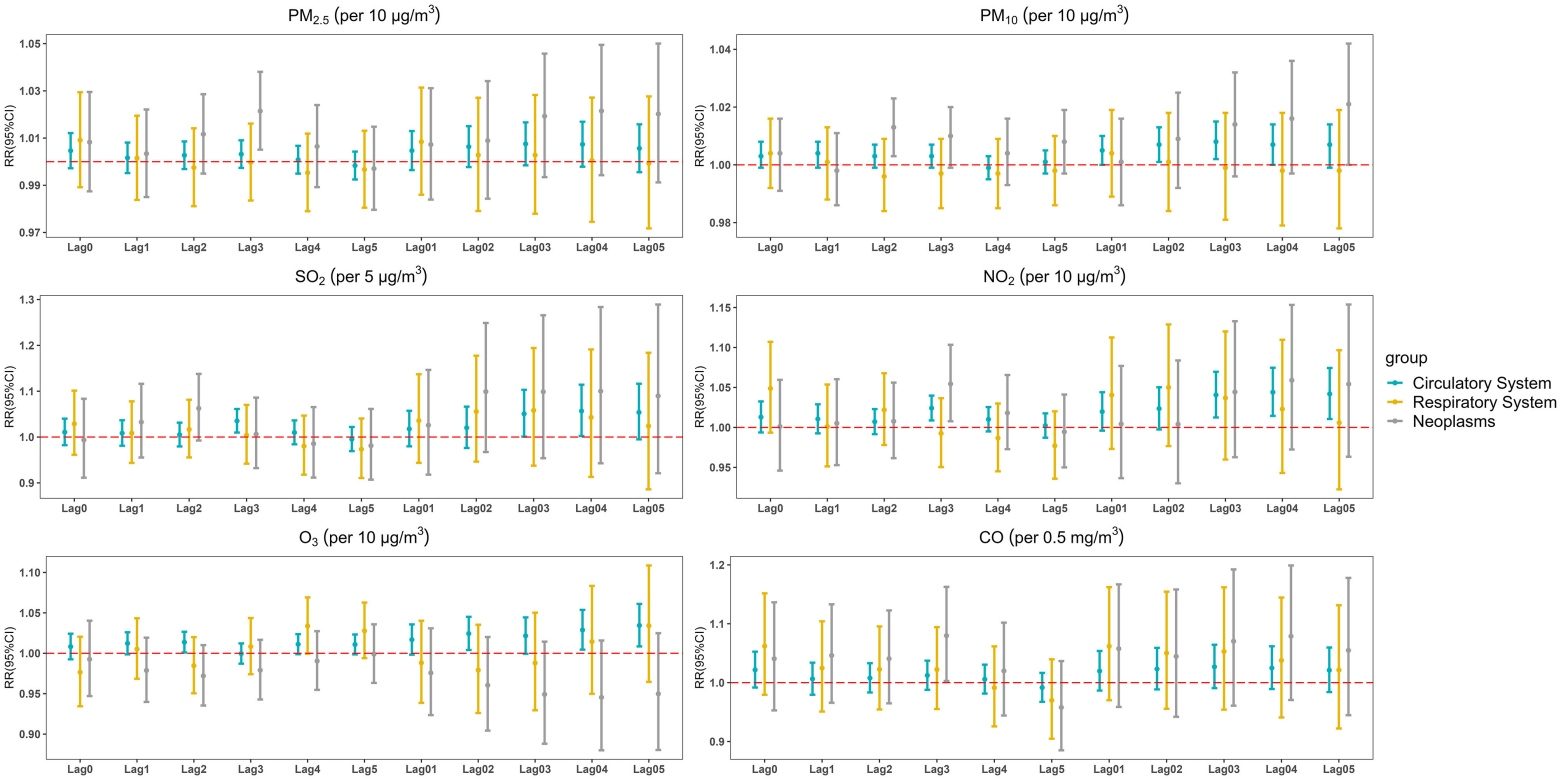
The estimated RRs with 95% CIs of cause-specific mortality associated with a 10 μg/m^3^ increase in air pollutant concentrations (5 μg/m^3^ in SO_2_, 0.5 mg/m^3^ in CO) at different lag days.

Figure 2 shows the relationship between air pollution and cause-specific mortality in different lag models. We found that these pollutants (PM_2.5_ and CO) only have significant positive associations with neoplasms disease mortality at lag 3, the RRs were 1.021 (95% CI: 1.005, 1.038) for PM_2.5_ and 1.080 (95% CI: 1.003, 1.163) for CO. Meanwhile, SO_2_ (lag 3, lag 03, and lag 04) and O_3_ (lag 2, lag 02, lag 04, and lag 05) were only associated with circulatory disease mortality, the RRs were 1.057 (95% CI: 1.002, 1.114) for SO_2_ (lag 04) and 1.034 (95% CI: 1.009, 1.061) for O_3_ (lag 05). Short-term exposure to NO_2_ had the significant impact on circulatory disease mortality in cumulative lag models and the RR was 1.044 (95% CI: 1.014, 1.075) in lag 04. The detailed results were shown in Supplementary Table 3.

**Figure 3:**
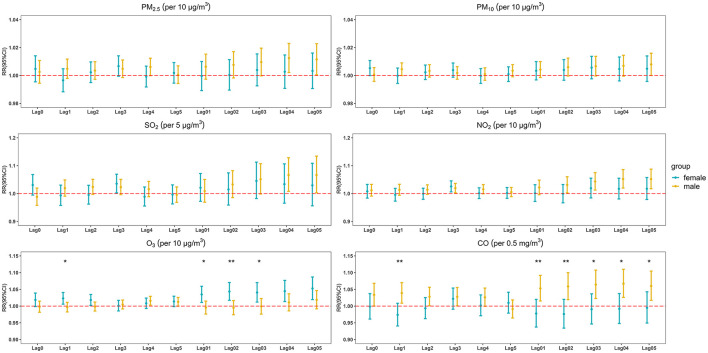
The estimated RRs with 95% CIs of daily non-accidental deaths in emergency department visits associated with a 10 μg/m^3^ increase in air pollutant concentrations (5 μg/m^3^ in SO_2_, 0.5 mg/m^3^ in CO) in sex-stratified analysis at different lag days (statistically significant for between-group difference *p < 0.05; **p < 0.01).

The results of the sex-specific analyses in different lag models are shown in Figure 3. Short-term exposure to CO and NO_2_ had the significant impact on males, and the RR values were 1.052 (95% CI: 1.020, 1.086) for NO_2_ at lag 04 and 1.067 (95% CI: 1.026, 1.110) for CO at lag 04 (the sex modification was statistically significant for CO). Meanwhile, the correlation between O_3_ and daily non-accidental deaths in emergency department visits was significantly positive in females, and the RR was 1.053 (95% CI: 1.019, 1.087) at lag 05 (the sex modification was statistically significant). The detailed results of stratified analyses by sex were in Supplementary Table 4.

**Figure 4:**
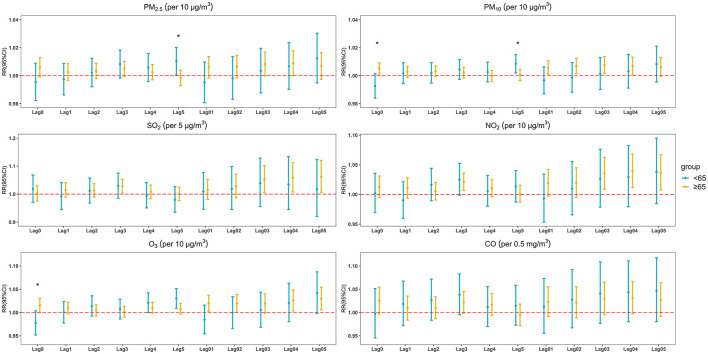
The estimated RRs with 95% CIs of non-accidental mortality in emergency department visits associated with a 10 μg/m^3^ increase in air pollutant concentrations (5 μg/m^3^ in SO_2_, 0.5 mg/m^3^ in CO) in age-stratified analysis at different lag days (statistically significant for between-group difference *p < 0.05).

The outcomes of the stratified analysis by age in different lag models are shown in Figure 4. Short-term exposure to the four air pollutants (PM_10_, SO_2_, NO_2_, and O_3_) had significant effects on people aged 65 years and older (the age modification was not statistically significant). The strongest effects observed in response to a unit increase in exposure were 1.008 (95% CI: 1.002, 1.014) for PM_10_ at lag 03, 1.062 (95% CI: 1.007, 1.121) for SO_2_ at lag 05, 1.040 (95% CI: 1.012, 1.068) for NO_2_ at lag 04, 1.030 (95% CI: 1.006, 1.054) for O_3_ at lag 05. Detailed outcomes of the age-specific stratified analysis are shown in Supplementary Table 5.

In sensitivity analysis, our findings were robust by adjusting the *dfs* to control for long-time trends (10–14 *df* per year), temperature (2–7 *df*), and relative humidity (2–7 *df* ; Supplementary Tables 6–8). After controlling for other pollutants, the short-term effects of air pollutants on non-accidental mortality in co-pollutant models are shown in Table 3 and Supplementary Table 9. The lag periods used in models were determined based on the maximum lag effect between non-accidental mortality and air pollution in single-pollutant models (lag 05 for PM_10_ and O_3_, lag 04 for other air pollutants). After adjusting for other pollutants in two-pollutant models, the positive associations between air pollutants and daily non-accidental deaths from emergency department visits remained significant, which demonstrated the relative robustness of the estimated effects. After adjusting for temperature of longer lag days (up to 28 days), the effect estimates changed slightly (Supplementary Table 10).

**Table 3:** The estimated RRs with 95% CIs for daily non-accidental deaths in emergency department visits associated with a 10 μg/m^3^ increment in air pollutant concentrations (5 μg/m^3^ in SO_2_, 0.5 mg/m^3^ in CO) in two-pollutant models.

**Two-pollutant models**	**Adjust for**	**RR (95% CI)**
PM_2.5_ (lag 04)	-	1.008 (1.001, 1.016)^a^
	+SO_2_	1.008 (1.000, 1.016)^a^
	+O_3_	1.009 (1.001, 1.017)^a^
PM_10_ (lag 05)	-	1.007 (1.001, 1.013)^a^
	+SO_2_	1.007 (1.001, 1.013)^a^
	+O_3_	1.007 (1.001, 1.013)^a^
	+CO	1.006 (0.999, 1.012)
SO_2_ (lag 04)	-	1.054 (1.009, 1.100)^a^
	+PM_2.5_	1.051 (1.003, 1.101)^a^
	+PM_10_	1.048 (1.002, 1.096)^a^
	+O_3_	1.054 (1.009, 1.101)^a^
	+CO	1.048 (1.001, 1.097)^a^
NO_2_ (lag 04)	-	1.038 (1.013, 1.063)^b^
	+O_3_	1.040 (1.015, 1.065)^b^
O_3_ (lag 05)	-	1.032 (1.011, 1.054)^b^
	+PM_2.5_	1.034 (1.013, 1.056)^b^
	+PM_10_	1.035 (1.013, 1.056)^b^
	+SO_2_	1.033 (1.012, 1.054)^b^
	+NO_2_	1.037 (1.016, 1.059)^b^
	+CO	1.035 (1.013, 1.056)^b^
CO (lag 04)	-	1.034 (1.004, 1.066)^a^
	+PM_10_	1.030 (0.999, 1.063)
	+SO_2_	1.034 (1.004, 1.066)^a^
	+O_3_	1.036 (1.005, 1.067)^a^

^a^*p* < 0.05.

^b^*p* < 0.01.

**Figure 5:**
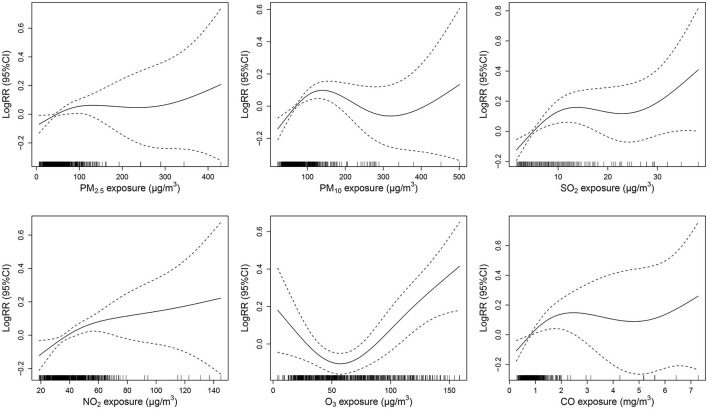
The exposure-response (E-R) curves for the association between six air pollutants and non-accidental mortality.

Figure 5 shows the E-R association between air pollutant levels (lag 05 for PM_10_ and O_3_, lag 04 for other air pollutants) and non-accidental mortality in emergency department visits. An approximate linear effect of NO_2_ was found for non-accidental mortality. Non-accidental morbidity monotonously increased with the concentrations of NO_2_. Meanwhile, the E-R curve for O_3_ was *U*-shaped and it showed potential risk within the concentration of < 30 or > 75 μg/m^3^.

## 4. Discussion

So far as we know, there are few studies examining the short-term effects of gaseous pollutants and particulate matter on non-accidental mortality based on data from emergency department visits in Beijing. Our data were obtained through the biggest emergency center in Beijing, which could more reasonably and accurately reflect the acute health effects from ambient pollution (26). Short-term exposure to air pollutants, especially gaseous pollutants, was demonstrated to have a positive relationship with non-accidental mortality. Meanwhile, short-term exposure to NO_2_, SO_2_, and O_3_ increased the risk of circulatory mortality. Additionally, stronger associations were presented in females for O_3_ while in males for CO. People aged 65 and over are strongly associated with ambient air pollution.

Gaseous pollutants had greater effects on non-accidental mortality than particulate matter in this study. In previous studies, such results were also found (31, 36). This may be related to a variety of factors including residents' awareness of self-protection, exposure levels, biological mechanisms, and population susceptibility. Gaseous pollutants are more likely to cause acute diseases, such as acute myocardial infarction, acute respiratory infections, and ischemic heart disease (37–39). Some studies have demonstrated that long-term exposure (e.g., several years) to particulate matter increases the risk of mortality to an even larger extent than exposure over a few days (40, 41). It has been shown that long-term exposure to PM_2.5_ is related to accelerated coronary artery calcification or increased the risk to plaque rupture (42). One study concluded that people had less awareness of protection against ozone when compared to particulate matter (43). The E-R associations of gaseous pollutant concentrations with non-accidental mortality were positive. It was interesting to note that the E-R curve for O_3_ was *U*-shaped, which showed a potential risk in the concentration range of < 30 or >75 μg/m^3^. Similar results were found in a study conducted in Delhi, India (44). This could be related to seasonal fluctuations in O_3_, with higher concentrations in summer. Synergy effects between temperature and O_3_ for non-accidental mortality are also biologically plausible (45, 46). The State Council of China issued the APCAP in 2013, but the annual average concentrations of NO_2_ and O_3_ in China have not changed significantly (47). This study indicates that emission control efforts for gaseous pollutants and people's awareness of protection should be strengthened in the future.

The relationship between short-term exposure to ambient gaseous pollution and non-accidental deaths has been extensively explored in developed countries. Similar to our research results, several previous meta-analyses based on North America and Europe investigated the relationship between ambient ozone and non-accidental mortality which demonstrated a statistically significant risk effect for non-accidental mortality (48, 49). There was a high positive association between most air pollutants (PM_2.5_, SO_2_, NO_2_, and CO) and daily mortality from diabetes in a study conducted in Montreal, Quebec, Canada (50). Similar outcomes related to non-accidental mortality have been discussed in China. A study conducted in Hefei, an inland city in China, showed that per 10 μg/m^3^ increment in SO_2_ (lag 03) and NO_2_ (lag 01) were significantly associated with 4.93% (95% CI: 1.94, 8.00), 2.11% (95% CI: 1.18, 3.05) increase of daily non-accidental deaths (15). Similar to our findings, a study exploring the acute effects of SO_2_ and NO_2_ on mortality found non-accidental mortality in Beijing raised 0.60% (95% CI: 0.26, 0.95) for every 10 μg/m^3^ increase in daily NO_2_ concentration (20). Meanwhile, a meta-analysis of the acute effects of ambient ozone on mortality in Chinese cities showed that the percent change for non-accidental mortality was 0.42% (95% CI: 0.32, 0.52) with an increase of 10 μg/m^3^ in the maximum 8-h average concentration of O_3_ (51). Variations in effect estimates are caused by many factors, including variation across the populations, various analysis strategy, and issues related to data quality and measurement error (50).

For cause-specific mortality, there was a significant association between short-term exposure to NO_2_, SO_2_ and O_3_ on circulatory disease mortality. The relationships of increased NO_2_, SO_2_, and O_3_ with increased cardiovascular mortality were also observed in exist evidence (51–54). The biological mechanisms contributing to the link of NO_2_, SO_2_, and O_3_ to circulatory disease mortality pathologies may involve complex processes. Increase in oxidant stress appears to be a broadly applicable mechanism, regardless of the type of pollutants that contributes to the adverse effects of air pollution. NO_2_, SO_2_, and O_3_ induce oxidative stress, thereby triggering inflammatory responses and gene activation, potentially leading to endothelial dysfunction, atherothrombotic alterations, metabolic dysregulation, and the development of cardiometabolic diseases (55–58). Researches have shown that O_3_ can lead to an imbalance in heart rate variability and an increased sensitivity to myocardial calcium load, which can trigger vascular and cardiac injury, ultimately manifesting as cardiovascular disease (59, 60). SO_2_ can reduce the permeability of red blood cells and lead to organ shortages, while short-term exposure to it can lead to a decreased in cardiac vagal control measures (61). NO_2_ is considered a proxy for traffic-related pollutants and is often found in conjunction with traffic noise, which is also associated with adverse cardiovascular health outcomes (62).

In stratified analysis, the effect estimates for CO were consistently higher in males, while the effect estimates for O_3_ were consistently higher in females. The sex modification was statistically significant for CO and O_3_. Several studies showed that males were more sensitive to air pollutants (32, 43, 63). The smoking rate for males is significantly higher than for females in China. Even after quitting smoking, impaired lung function may not recover to normal quickly, and may have synergistic effects with air pollution (64). Meanwhile, some studies have suggested that the increased susceptibility of females to the adverse effects of O_3_, which could be related to differential regulation of the lung immune response (65). The reasons for gender vulnerability to air pollution were not clear and need further investigation. In the stratified analyses by age, the association between short-term exposure to air pollutants and non-accidental deaths seemed to be evident in older people. Several foreign studies have also reported increased susceptibility in older people (66, 67). Studies confirm that certain physiological regulatory functions diminish with age, which may lead to an increased health risk associated with those older than 65 years (68). Some studies have indicated that current air quality guidelines are designed to protect the general population but not enough to protect older people (69). Therefore, we call for more studies to investigate the susceptibility of older people to air pollutant concentrations in different countries or different regions with the aim of determining whether this finding can be generalized across different climatic and air pollution characteristics.

Several limitations exist within this study that should be considered. First, we use data on air pollution from the average of fixed-site air quality–monitoring stations to represent exposure at the individual level, which can lead to exposure misclassification. Secondly, this study fails to control for social and demographic factors (e.g., education level and socioeconomic status) which could influence the demographic composition and mortality (70, 71). Thirdly, this is an ecological research limiting causal inference. This needs to be addressed by further toxicological or epidemiological studies. Finally, it was a single-center study with limited study time. Therefore, we encourage further large-scale studies, especially in developing countries.

## 5. Conclusion

Our findings demonstrated that short-term exposure to ambient pollutants, especially gaseous pollutants, increased the risk of non-accidental mortality in emergency department visits to Beijing. Additionally, stronger associations were presented in females for O_3_ while in males for CO. People aged 65 and over are strongly associated with ambient air pollution. The study provides health authorities with critical information on the acute adverse effects of air pollution, which will contribute to the development of protective measures in China.

## Data availability statement

The data that support the findings of this study are available from the corresponding authors, upon reasonable request.

## Author contributions

SW and YZ: conceptualization, formal analysis, and writing—original draft preparation. XL: software and writing—review and editing. JZ: methodology. NZ: validation and visualization. YG: validation. JC, YLi, and YLy: data curation. ZC: writing—original draft preparation. CL: project administration. JG: software and funding acquisition. WZ: methodology and resources. JM: investigation, resources, and supervision. All authors contributed to the article and approved the submitted version.
