# Supplementary material for: Effect of short-term exposure to ambient air pollutants on non-accidental mortality in emergency department visits: a time-series study
Source: Front Public Health. 2023 Jun 30;11:1208514. doi: 10.3389/fpubh.2023.1208514 (PMC10348907; doi:10.3389/fpubh.2023.1208514)
Supplement: Supplementary file 1 [file Table_1.docx]

Effect of Short-Term Exposure to ambient air pollutants on non-accidental mortality in emergency department visits: A time-series study

**Supplemental Table 1** Descriptive characteristics for annual non-accidental deaths in Beijing Red Cross Emergency Rescue Center from 2017 to 2018.

| Characteristics | 2017(%) | 2018(%) | Total |
| --- | --- | --- | --- |
| Total (A00-R99) | 3916 | 4760 | 8676 |
| Sex | | | |
| Male | 2316 (59.14) | 2748(57.73) | 5064 (58.37) |
| Female | 1600 (40.86) | 2012 (42.27) | 3612 (41.63) |
| Age | | | |
| <65 | 915 (23.37) | 1071 (22.50) | 1986 (22.89) |
| ≥65 | 3001 (76.63) | 3689 (77.50) | 6690 (77.11) |
| Causes of death | | | |
| Neoplasms(C00-D48) | 312 (7.97) | 347 (7.29) | 659 (7.60) |
| Respiratory system (J00-J99) | 345 (8.81) | 372 (7.82) | 717 (8.26) |
| Circulatory system (I00-I99) | 2734 (69.82) | 3064 (64.37) | 5798 (66.83) |

**Supplemental Table 2** Correlations between daily air pollutant concentrations and meteorological variables

| Variable | PM_10_ | PM_2.5_ | SO_2_ | NO_2_ | CO | O_3_ | Temperature | Relative humidity |
| --- | --- | --- | --- | --- | --- | --- | --- | --- |
| PM_10_ | 1 | 0.78^b^ | 0.52^b^ | 0.61^b^ | 0.58^b^ | 0.04 | 0.06 | 0.04 |
| PM_2.5_ |  | 1 | 0.46^b^ | 0.65^b^ | 0.85^b^ | -0.03 | 0.09^a^ | 0.40^b^ |
| SO_2_ |  |  | 1 | 0.61^b^ | 0.55^b^ | -0.29^b^ | -0.45^b^ | -0.27^b^ |
| NO_2_ |  |  |  | 1 | 0.69^b^ | -0.47^b^ | -0.25^b^ | 0.12^b^ |
| CO |  |  |  |  | 1 | -0.23^b^ | -0.05 | 0.45^b^ |
| O_3_ |  |  |  |  |  | 1 | 0.74^b^ | 0.08^a^ |
| Temperature |  |  |  |  |  |  | 1 | 0.50^b^ |
| Relative humidity |  |  |  |  |  |  |  | 1 |

^a^ *p*<0.05; ^b^ *p*<0.01

**Supplemental Table 3** The estimated RRs with 95% CIs of cause-specific mortality associated with a 10 μg/m^3^ increase in air pollutant concentrations (5 μg/m^3^ in SO_2_, 0.5 mg/m^3^ in CO) at different lag days

| Air pollutants | Lag days | The estimated RRs with 95% CIs | | |
| --- | --- | --- | --- | --- |
|  |  | Circulatory system | Respiratory system | Neoplasms |
| PM_2.5_ | Lag 0 | 1.005(0.997,1.012) | 1.009(0.989,1.029) | 1.008(0.987,1.030) |
|  | Lag 1 | 1.002(0.995,1.008) | 1.001(0.984,1.019) | 1.003(0.985,1.022) |
|  | Lag 2 | 1.003(0.997,1.009) | 0.998(0.981,1.014) | 1.012(0.995,1.029) |
|  | Lag 3 | 1.003(0.997,1.009) | 1.000(0.984,1.016) | 1.021(1.005,1.038)^b^ |
|  | Lag 4 | 1.001(0.995,1.007) | 0.995(0.979,1.012) | 1.006(0.989,1.024) |
|  | Lag 5 | 0.998(0.992,1.004) | 0.997(0.980,1.013) | 0.997(0.980,1.015) |
|  | Lag 01 | 1.005(0.996,1.013) | 1.008(0.986,1.031) | 1.007(0.984,1.031) |
|  | Lag 02 | 1.006(0.998,1.015) | 1.003(0.979,1.027) | 1.009(0.984,1.034) |
|  | Lag 03 | 1.008(0.998,1.017) | 1.003(0.978,1.028) | 1.019(0.993,1.046) |
|  | Lag 04 | 1.007(0.998,1.017) | 1.000(0.975,1.027) | 1.022(0.994,1.049) |
|  | Lag 05 | 1.006(0.996,1.016) | 0.999(0.972,1.028) | 1.020(0.991,1.050) |
| PM_10_ | Lag 0 | 1.003(0.999,1.008) | 1.004(0.992,1.016) | 1.004(0.991,1.016) |
|  | Lag 1 | 1.004(0.999,1.008) | 1.001(0.988,1.013) | 0.998(0.986,1.011) |
|  | Lag 2 | 1.003(0.999,1.007) | 0.996(0.984,1.009) | 1.013(1.003,1.023)^a^ |
|  | Lag 3 | 1.003(0.999,1.007) | 0.997(0.985,1.009) | 1.010(0.999,1.020) |
|  | Lag 4 | 0.999(0.995,1.003) | 0.997(0.985,1.009) | 1.004(0.993,1.016) |
|  | Lag 5 | 1.001(0.997,1.005) | 0.998(0.986,1.010) | 1.008(0.997,1.019) |
|  | Lag 01 | 1.005(1.000,1.010) | 1.004(0.989,1.019) | 1.001(0.986,1.016) |
|  | Lag 02 | 1.007(1.001,1.013)^a^ | 1.001(0.984,1.018) | 1.009(0.992,1.025) |
|  | Lag 03 | 1.008(1.002,1.015)^a^ | 0.999(0.981,1.018) | 1.014(0.996,1.032) |
|  | Lag 04 | 1.007(1.000,1.014)^a^ | 0.998(0.979,1.018) | 1.016(0.997,1.036) |
|  | Lag 05 | 1.007(0.999,1.014) | 0.998(0.978,1.019) | 1.021(1.000,1.042)^a^ |
| SO_2_ | Lag 0 | 1.011(0.982,1.040) | 1.029(0.961,1.101) | 0.994(0.911,1.083) |
|  | Lag 1 | 1.008(0.981,1.037) | 1.009(0.944,1.078) | 1.033(0.955,1.116) |
|  | Lag 2 | 1.005(0.980,1.031) | 1.017(0.956,1.081) | 1.063(0.992,1.138) |
|  | Lag 3 | 1.035(1.010,1.061)^b^ | 1.004(0.942,1.070) | 1.006(0.932,1.086) |
|  | Lag 4 | 1.010(0.984,1.036) | 0.980(0.918,1.047) | 0.985(0.912,1.065) |
|  | Lag 5 | 0.995(0.969,1.022) | 0.973(0.911,1.040) | 0.981(0.907,1.061) |
|  | Lag 01 | 1.018(0.980,1.057) | 1.036(0.944,1.137) | 1.026(0.918,1.146) |
|  | Lag 02 | 1.020(0.976,1.066) | 1.056(0.946,1.177) | 1.099(0.967,1.249) |
|  | Lag 03 | 1.051(1.001,1.103)^a^ | 1.058(0.938,1.194) | 1.099(0.954,1.266) |
|  | Lag 04 | 1.057(1.002,1.114)^a^ | 1.043(0.913,1.191) | 1.100(0.943,1.284) |
|  | Lag 05 | 1.054(0.995,1.116) | 1.024(0.886,1.184) | 1.090(0.921,1.289) |
| NO_2_ | Lag 0 | 1.013(0.994,1.033) | 1.049(0.993,1.107) | 1.001(0.946,1.059) |
|  | Lag 1 | 1.011(0.993,1.029) | 1.001(0.951,1.054) | 1.005(0.953,1.060) |
|  | Lag 2 | 1.007(0.992,1.023) | 1.022(0.978,1.068) | 1.008(0.962,1.056) |
|  | Lag 3 | 1.024(1.009,1.040)^b^ | 0.992(0.950,1.037) | 1.054(1.008,1.103)^a^ |
|  | Lag 4 | 1.010(0.995,1.025) | 0.987(0.945,1.030) | 1.018(0.973,1.066) |
|  | Lag 5 | 1.002(0.987,1.018) | 0.977(0.936,1.020) | 0.994(0.950,1.041) |
|  | Lag 01 | 1.020(0.996,1.044) | 1.040(0.973,1.113) | 1.004(0.936,1.077) |
|  | Lag 02 | 1.024(0.997,1.050) | 1.050(0.977,1.129) | 1.004(0.930,1.084) |
|  | Lag 03 | 1.041(1.012,1.070)^b^ | 1.037(0.960,1.120) | 1.044(0.963,1.133) |
|  | Lag 04 | 1.044(1.014,1.075)^b^ | 1.023(0.943,1.110) | 1.059(0.972,1.153) |
|  | Lag 05 | 1.042(1.011,1.074)^b^ | 1.006(0.922,1.097) | 1.054(0.963,1.154) |
| CO | Lag 0 | 1.022(0.992,1.053) | 1.062(0.979,1.152) | 1.041(0.953,1.136) |
|  | Lag 1 | 1.006(0.979,1.034) | 1.025(0.951,1.104) | 1.046(0.966,1.133) |
|  | Lag 2 | 1.008(0.983,1.033) | 1.023(0.954,1.096) | 1.041(0.965,1.123) |
|  | Lag 3 | 1.012(0.988,1.037) | 1.022(0.955,1.094) | 1.080(1.003,1.163)^a^ |
|  | Lag 4 | 1.006(0.981,1.031) | 0.991(0.926,1.062) | 1.020(0.944,1.102) |
|  | Lag 5 | 0.992(0.967,1.017) | 0.970(0.905,1.040) | 0.958(0.885,1.037) |
|  | Lag 01 | 1.020(0.987,1.054) | 1.062(0.970,1.162) | 1.058(0.959,1.167) |
|  | Lag 02 | 1.023(0.988,1.059) | 1.050(0.956,1.155) | 1.045(0.942,1.158) |
|  | Lag 03 | 1.027(0.991,1.064) | 1.053(0.954,1.162) | 1.070(0.961,1.192) |
|  | Lag 04 | 1.025(0.989,1.062) | 1.038(0.941,1.145) | 1.079(0.970,1.199) |
|  | Lag 05 | 1.021(0.984,1.060) | 1.021(0.922,1.132) | 1.055(0.945,1.178) |
| O_3_ | Lag 0 | 1.008(0.993,1.024) | 0.977(0.934,1.020) | 0.993(0.947,1.040) |
|  | Lag 1 | 1.012(0.999,1.026) | 1.005(0.968,1.043) | 0.979(0.940,1.019) |
|  | Lag 2 | 1.014(1.001,1.027)^a^ | 0.985(0.951,1.020) | 0.972(0.935,1.010) |
|  | Lag 3 | 1.000(0.987,1.012) | 1.008(0.974,1.044) | 0.979(0.943,1.017) |
|  | Lag 4 | 1.011(0.999,1.024) | 1.034(1.000,1.069) | 0.990(0.955,1.027) |
|  | Lag 5 | 1.011(0.999,1.023) | 1.028(0.994,1.063) | 0.999(0.963,1.036) |
|  | Lag 01 | 1.017(0.998,1.036) | 0.988(0.939,1.040) | 0.976(0.924,1.031) |
|  | Lag 02 | 1.024(1.004,1.045)^a^ | 0.979(0.926,1.035) | 0.961(0.904,1.020) |
|  | Lag 03 | 1.022(0.999,1.044) | 0.988(0.930,1.050) | 0.949(0.888,1.014) |
|  | Lag 04 | 1.029(1.004,1.054)^a^ | 1.014(0.950,1.083) | 0.945(0.880,1.016) |
|  | Lag 05 | 1.034(1.009,1.061)^b^ | 1.034(0.965,1.109) | 0.950(0.880,1.025) |

^a^ *p*<0.05; ^b^ *p*<0.01

**Supplemental Table 4** The estimated RRs with 95% CIs of daily non-accidental deaths in emergency department visits associated with a 10 μg/m^3^ increase in air pollutant concentrations (5 μg/m^3^ in SO_2_, 0.5 mg/m^3^ in CO) in sex-stratified analysis at different lag days

| Air pollutants | Lag days | The estimated RRs with 95% CIs | | *Z** |
| --- | --- | --- | --- | --- |
|  |  | Male | Female |  |
| PM_2.5_ | Lag 0 | 1.003(0.994,1.011) | 1.005(0.995,1.014) | -0.345 |
|  | Lag 1 | 1.005(0.998,1.012) | 0.997(0.988,1.005) | 1.506 |
|  | Lag 2 | 1.004(0.997,1.010) | 1.002(0.995,1.010) | 0.253 |
|  | Lag 3 | 1.005(0.999,1.011) | 1.007(0.999,1.014) | -0.390 |
|  | Lag 4 | 1.006(1.000,1.012) | 0.999(0.992,1.007) | 1.385 |
|  | Lag 5 | 1.001(0.994,1.007) | 1.002(0.995,1.009) | -0.260 |
|  | Lag 01 | 1.006(0.997,1.015) | 1.000(0.989,1.010) | 0.960 |
|  | Lag 02 | 1.008(0.998,1.017) | 1.000(0.990,1.011) | 0.979 |
|  | Lag 03 | 1.010(1.000,1.020) | 1.004(0.993,1.015) | 0.731 |
|  | Lag 04 | 1.012(1.002,1.023)^a^ | 1.003(0.991,1.015) | 1.211 |
|  | Lag 05 | 1.012(1.001,1.023)^a^ | 1.003(0.991,1.016) | 0.965 |
| PM_10_ | Lag 0 | 1.001(0.996,1.006) | 1.005(1.000,1.011) | -1.231 |
|  | Lag 1 | 1.004(1.000,1.009) | 1.000(0.994,1.005) | 1.279 |
|  | Lag 2 | 1.003(0.999,1.008) | 1.002(0.997,1.007) | 0.287 |
|  | Lag 3 | 1.002(0.997,1.006) | 1.004(0.999,1.009) | -0.582 |
|  | Lag 4 | 1.001(0.997,1.006) | 1.000(0.994,1.005) | 0.391 |
|  | Lag 5 | 1.003(0.999,1.008) | 1.001(0.996,1.006) | 0.702 |
|  | Lag 01 | 1.004(0.999,1.010) | 1.003(0.997,1.010) | 0.198 |
|  | Lag 02 | 1.006(1.000,1.012) | 1.004(0.997,1.011) | 0.409 |
|  | Lag 03 | 1.007(1.000,1.014) | 1.006(0.998,1.014) | 0.185 |
|  | Lag 04 | 1.007(1.000,1.014) | 1.005(0.996,1.013) | 0.403 |
|  | Lag 05 | 1.008(1.000,1.016)^a^ | 1.005(0.996,1.014) | 0.505 |
| SO_2_ | Lag 0 | 0.988(0.958,1.020) | 1.031(0.995,1.068) | -1.736 |
|  | Lag 1 | 1.019(0.991,1.049) | 0.993(0.957,1.030) | 1.094 |
|  | Lag 2 | 1.024(0.997,1.051) | 0.995(0.962,1.030) | 1.277 |
|  | Lag 3 | 1.023(0.996,1.051) | 1.036(1.003,1.069)^a^ | -0.565 |
|  | Lag 4 | 1.016(0.989,1.044) | 0.989(0.955,1.024) | 1.201 |
|  | Lag 5 | 0.996(0.968,1.024) | 0.996(0.963,1.031) | -0.028 |
|  | Lag 01 | 1.009(0.969,1.051) | 1.021(0.972,1.072) | -0.364 |
|  | Lag 02 | 1.033(0.985,1.082) | 1.015(0.959,1.074) | 0.464 |
|  | Lag 03 | 1.051(0.998,1.107) | 1.045(0.982,1.113) | 0.138 |
|  | Lag 04 | 1.066(1.008,1.128)^a^ | 1.034(0.966,1.107) | 0.691 |
|  | Lag 05 | 1.067(1.003,1.134)^a^ | 1.030(0.956,1.109) | 0.720 |
| NO_2_ | Lag 0 | 1.012(0.991,1.033) | 1.008(0.984,1.033) | 0.229 |
|  | Lag 1 | 1.013(0.994,1.033) | 0.996(0.973,1.019) | 1.158 |
|  | Lag 2 | 1.014(0.997,1.031) | 0.999(0.979,1.019) | 1.116 |
|  | Lag 3 | 1.020(1.003,1.036)^a^ | 1.026(1.006,1.045)^a^ | -0.453 |
|  | Lag 4 | 1.015(0.999,1.032) | 1.001(0.982,1.021) | 1.095 |
|  | Lag 5 | 1.005(0.989,1.022) | 1.002(0.983,1.022) | 0.248 |
|  | Lag 01 | 1.022(0.996,1.048) | 1.001(0.972,1.032) | 0.999 |
|  | Lag 02 | 1.031(1.003,1.060)^a^ | 0.999(0.967,1.032) | 1.430 |
|  | Lag 03 | 1.043(1.013,1.075)^b^ | 1.019(0.984,1.055) | 1.000 |
|  | Lag 04 | 1.052(1.020,1.086)^b^ | 1.017(0.981,1.055) | 1.381 |
|  | Lag 05 | 1.052(1.017,1.088)^b^ | 1.017(0.979,1.058) | 1.282 |
| CO | Lag 0 | 1.034(1.000,1.068)^a^ | 0.999(0.961,1.037) | 1.340 |
|  | Lag 1 | 1.039(1.009,1.070)^a^ | 0.974(0.941,1.008) | 2.779^b^ |
|  | Lag 2 | 1.028(1.001,1.056)^a^ | 0.993(0.962,1.025) | 1.620 |
|  | Lag 3 | 1.028(1.001,1.056)^a^ | 1.022(0.991,1.054) | 0.278 |
|  | Lag 4 | 1.026(0.999,1.054) | 1.002(0.971,1.033) | 1.158 |
|  | Lag 5 | 0.991(0.964,1.018) | 1.010(0.979,1.042) | -0.885 |
|  | Lag 01 | 1.053(1.015,1.092)^b^ | 0.978(0.937,1.020) | 2.599^b^ |
|  | Lag 02 | 1.059(1.019,1.100)^b^ | 0.976(0.934,1.020) | 2.731^b^ |
|  | Lag 03 | 1.064(1.023,1.108)^b^ | 0.991(0.947,1.037) | 2.327^a^ |
|  | Lag 04 | 1.067(1.026,1.110)^b^ | 0.992(0.948,1.037) | 2.402^a^ |
|  | Lag 05 | 1.060(1.017,1.105)^b^ | 0.995(0.949,1.043) | 1.989^a^ |
| O_3_ | Lag 0 | 0.998(0.982,1.015) | 1.018(0.998,1.039) | -1.532 |
|  | Lag 1 | 0.997(0.983,1.011) | 1.023(1.006,1.041)^b^ | -2.294^a^ |
|  | Lag 2 | 0.999(0.985,1.012) | 1.018(1.002,1.035)^a^ | -1.836 |
|  | Lag 3 | 1.005(0.991,1.018) | 1.001(0.986,1.017) | 0.326 |
|  | Lag 4 | 1.016(1.003,1.029)^a^ | 1.008(0.993,1.024) | 0.729 |
|  | Lag 5 | 1.013(1.000,1.026) | 1.014(0.998,1.030) | -0.109 |
|  | Lag 01 | 0.995(0.976,1.015) | 1.035(1.011,1.059)^b^ | -2.503^a^ |
|  | Lag 02 | 0.995(0.974,1.016) | 1.044(1.017,1.070)^b^ | -2.818^b^ |
|  | Lag 03 | 0.999(0.976,1.023) | 1.041(1.012,1.070)^b^ | -2.197^a^ |
|  | Lag 04 | 1.011(0.986,1.037) | 1.045(1.014,1.077)^b^ | -1.640 |
|  | Lag 05 | 1.019(0.992,1.046) | 1.053(1.019,1.087)^b^ | -1.538 |

*The RRs and *Z*-values calculated from the two categories of effect estimates and standard errors were used to test the statistical significance of each subgroup’s difference

^a^ *p*<0.05; ^b^ *p*<0.01

**Supplemental Table 5** The estimated RRs with 95% CIs of non-accidental mortality in emergency department visits associated with a 10 μg/m^3^ increase in air pollutant concentrations (5 μg/m^3^ in SO_2_, 0.5 mg/m^3^ in CO) in age-stratified analysis at different lag days

| Air pollutants | Lag days | The estimated RRs with 95% CIs | | *Z** |
| --- | --- | --- | --- | --- |
|  |  | ＜65 | ≥65 |  |
| PM_2.5_ | Lag 0 | 0.995(0.982,1.009) | 1.006(0.999,1.013) | -1.380 |
|  | Lag 1 | 0.997(0.986,1.009) | 1.003(0.997,1.009) | -0.792 |
|  | Lag 2 | 1.002(0.992,1.012) | 1.003(0.998,1.009) | -0.196 |
|  | Lag 3 | 1.008(0.998,1.018) | 1.005(0.999,1.010) | 0.586 |
|  | Lag 4 | 1.006(0.996,1.016) | 1.003(0.997,1.008) | 0.566 |
|  | Lag 5 | 1.010(1.001,1.020)^a^ | 0.998(0.993,1.004) | 2.084^a^ |
|  | Lag 01 | 0.995(0.981,1.010) | 1.006(0.998,1.014) | -1.287 |
|  | Lag 02 | 0.998(0.983,1.014) | 1.006(0.998,1.015) | -0.937 |
|  | Lag 03 | 1.004(0.988,1.020) | 1.008(1.000,1.017) | -0.530 |
|  | Lag 04 | 1.007(0.990,1.024) | 1.009(1.000,1.018) | -0.214 |
|  | Lag 05 | 1.012(0.995,1.030) | 1.007(0.997,1.016) | 0.539 |
| PM_10_ | Lag 0 | 0.993(0.984,1.001) | 1.005(1.001,1.009)^a^ | -2.564^a^ |
|  | Lag 1 | 1.002(0.994,1.009) | 1.003(0.999,1.007) | -0.236 |
|  | Lag 2 | 1.002(0.995,1.009) | 1.003(0.999,1.007) | -0.263 |
|  | Lag 3 | 1.004(0.997,1.011) | 1.002(0.998,1.006) | 0.541 |
|  | Lag 4 | 1.003(0.996,1.010) | 1.000(0.996,1.004) | 0.667 |
|  | Lag 5 | 1.009(1.002,1.015)^a^ | 1.000(0.997,1.004) | 2.067^a^ |
|  | Lag 01 | 0.997(0.987,1.006) | 1.006(1.001,1.011)^a^ | -1.669 |
|  | Lag 02 | 0.999(0.988,1.009) | 1.007(1.001,1.012)^a^ | -1.344 |
|  | Lag 03 | 1.001(0.990,1.013) | 1.008(1.002,1.014)^a^ | -0.937 |
|  | Lag 04 | 1.003(0.991,1.015) | 1.007(1.001,1.013)^a^ | -0.536 |
|  | Lag 05 | 1.008(0.995,1.021) | 1.006(0.999,1.013) | 0.269 |
| SO_2_ | Lag 0 | 1.018(0.971,1.068) | 1.002(0.976,1.030) | 0.559 |
|  | Lag 1 | 0.992(0.945,1.041) | 1.014(0.989,1.041) | -0.802 |
|  | Lag 2 | 1.012(0.968,1.057) | 1.013(0.989,1.037) | -0.050 |
|  | Lag 3 | 1.029(0.985,1.075) | 1.028(1.004,1.052)^a^ | 0.041 |
|  | Lag 4 | 0.995(0.951,1.042) | 1.008(0.984,1.033) | -0.481 |
|  | Lag 5 | 0.980(0.935,1.027) | 1.001(0.977,1.025) | -0.771 |
|  | Lag 01 | 1.009(0.945,1.077) | 1.016(0.981,1.053) | -0.182 |
|  | Lag 02 | 1.019(0.945,1.099) | 1.028(0.987,1.071) | -0.197 |
|  | Lag 03 | 1.039(0.956,1.129) | 1.053(1.006,1.102)^a^ | -0.281 |
|  | Lag 04 | 1.035(0.945,1.134) | 1.059(1.008,1.113)^a^ | -0.431 |
|  | Lag 05 | 1.017(0.920,1.123) | 1.062(1.007,1.121)^a^ | -0.755 |
| NO_2_ | Lag 0 | 1.002(0.970,1.036) | 1.013(0.995,1.031) | -0.556 |
|  | Lag 1 | 0.990(0.960,1.021) | 1.011(0.994,1.028) | -1.143 |
|  | Lag 2 | 1.016(0.989,1.044) | 1.005(0.991,1.020) | 0.702 |
|  | Lag 3 | 1.025(0.999,1.052) | 1.021(1.007,1.036)^b^ | 0.253 |
|  | Lag 4 | 1.006(0.980,1.032) | 1.011(0.996,1.025) | -0.321 |
|  | Lag 5 | 1.013(0.987,1.040) | 1.001(0.987,1.016) | 0.801 |
|  | Lag 01 | 0.993(0.953,1.034) | 1.019(0.997,1.042) | -1.098 |
|  | Lag 02 | 1.010(0.966,1.056) | 1.020(0.995,1.045) | -0.390 |
|  | Lag 03 | 1.026(0.979,1.076) | 1.035(1.009,1.062)^b^ | -0.315 |
|  | Lag 04 | 1.030(0.979,1.082) | 1.040(1.012,1.068)^b^ | -0.337 |
|  | Lag 05 | 1.038(0.985,1.095) | 1.037(1.007,1.067)^a^ | 0.045 |
| CO | Lag 0 | 0.997(0.946,1.051) | 1.025(0.997,1.055) | -0.907 |
|  | Lag 1 | 1.019(0.972,1.067) | 1.009(0.984,1.035) | 0.338 |
|  | Lag 2 | 1.027(0.983,1.072) | 1.010(0.987,1.034) | 0.658 |
|  | Lag 3 | 1.038(0.996,1.083) | 1.022(0.998,1.045) | 0.661 |
|  | Lag 4 | 1.012(0.970,1.056) | 1.017(0.994,1.041) | -0.212 |
|  | Lag 5 | 1.015(0.973,1.059) | 0.995(0.972,1.018) | 0.818 |
|  | Lag 01 | 1.013(0.955,1.073) | 1.023(0.991,1.055) | -0.302 |
|  | Lag 02 | 1.028(0.967,1.092) | 1.022(0.989,1.056) | 0.171 |
|  | Lag 03 | 1.041(0.977,1.109) | 1.030(0.995,1.065) | 0.288 |
|  | Lag 04 | 1.043(0.980,1.111) | 1.032(0.997,1.067) | 0.317 |
|  | Lag 05 | 1.047(0.981,1.118) | 1.027(0.992,1.064) | 0.495 |
| O_3_ | Lag 0 | 0.977(0.952,1.004) | 1.015(1.001,1.030)^a^ | -2.455^a^ |
|  | Lag 1 | 1.000(0.978,1.024) | 1.010(0.997,1.023) | -0.700 |
|  | Lag 2 | 1.014(0.993,1.036) | 1.005(0.993,1.016) | 0.754 |
|  | Lag 3 | 1.007(0.986,1.029) | 1.002(0.990,1.014) | 0.420 |
|  | Lag 4 | 1.021(1.000,1.042)^a^ | 1.010(0.999,1.022) | 0.878 |
|  | Lag 5 | 1.030(1.009,1.051)^b^ | 1.008(0.997,1.020) | 1.809 |
|  | Lag 01 | 0.985(0.954,1.016) | 1.020(1.002,1.037)^a^ | -1.916 |
|  | Lag 02 | 0.999(0.966,1.034) | 1.019(1.001,1.039)^a^ | -1.005 |
|  | Lag 03 | 1.005(0.969,1.044) | 1.019(0.999,1.040) | -0.632 |
|  | Lag 04 | 1.021(0.980,1.063) | 1.026(1.003,1.049)^a^ | -0.215 |
|  | Lag 05 | 1.042(0.998,1.087) | 1.030(1.006,1.054)^a^ | 0.464 |

*The RRs and *Z*-values calculated from the two categories of effect estimates and standard errors were used to test the statistical significance of each subgroup’s difference

^a^ *p*<0.05; ^b^ *p*<0.01

**Supplemental Table 6** Sensitivity analyses for effect estimates of air pollution on non-accidental mortality by changing degrees of freedom (dfs) to control for time trend (10–14)

| Variable | dfs | The estimated RRs with 95% CIs |
| --- | --- | --- |
| PM_2.5_ (lag 04) | 10 | 1.009(1.001 ,1.017)^a^ |
|  | 11 | 1.009(1.001 ,1.017)^a^ |
|  | 12 | 1.008(1.001, 1.016)^a^ |
|  | 13 | 1.009(1.001, 1.017)^a^ |
|  | 14 | 1.009(1.000, 1.017)^a^ |
| PM_10_ (lag 05) | 10 | 1.006(1.000, 1.012)^a^ |
|  | 11 | 1.007(1.001, 1.013)^a^ |
|  | 12 | 1.007(1.001, 1.013)^a^ |
|  | 13 | 1.007(1.001, 1.013)^a^ |
|  | 14 | 1.006(1.000, 1.013)^a^ |
| SO_2_ (lag 04) | 10 | 1.044(1.001, 1.088)^a^ |
|  | 11 | 1.043(1.000, 1.089) |
|  | 12 | 1.054(1.009, 1.100)^a^ |
|  | 13 | 1.055(1.011, 1.102)^a^ |
|  | 14 | 1.050(1.006, 1.096)^a^ |
| NO_2_ (lag 04) | 10 | 1.039(1.016, 1.064)^b^ |
|  | 11 | 1.036(1.012, 1.060)^b^ |
|  | 12 | 1.038(1.013, 1.063)^b^ |
|  | 13 | 1.039(1.014, 1.065)^b^ |
|  | 14 | 1.040(1.013, 1.067)^b^ |
| O_3_ (lag 05) | 10 | 1.032(1.012, 1.053)^b^ |
|  | 11 | 1.030(1.008, 1.052)^b^ |
|  | 12 | 1.032(1.011, 1.054)^b^ |
|  | 13 | 1.029(1.007, 1.052)^b^ |
|  | 14 | 1.037(1.014, 1.060)^b^ |
| CO (lag 04) | 10 | 1.032(1.003, 1.062)^a^ |
|  | 11 | 1.034(1.004, 1.065)^a^ |
|  | 12 | 1.034(1.004, 1.066)^a^ |
|  | 13 | 1.032(1.001, 1.064)^a^ |
|  | 14 | 1.030(0.998, 1.063) |

^a^ *p*<0.05; ^b^ *p*<0.01

**Supplemental Table 7** Sensitivity analyses for effect estimates of air pollution on non-accidental mortality by changing degrees of freedom (dfs) to control for temperature (2–7)

| Variable | dfs | The estimated RRs with 95% CIs |
| --- | --- | --- |
| PM_2.5_ (lag 04) | 2 | 1.009(1.001, 1.017)^a^ |
|  | 3 | 1.009(1.001, 1.017)^a^ |
|  | 4 | 1.009(1.001, 1.017)^a^ |
|  | 5 | 1.009(1.001, 1.016)^a^ |
|  | 6 | 1.008(1.001, 1.016)^a^ |
|  | 7 | 1.008(1.001, 1.016)^a^ |
| PM_10_ (lag 05) | 2 | 1.006(1.000, 1.012)^a^ |
|  | 3 | 1.006(1.000, 1.012)^a^ |
|  | 4 | 1.007(1.001, 1.013)^a^ |
|  | 5 | 1.007(1.001, 1.013)^a^ |
|  | 6 | 1.007(1.001, 1.013)^a^ |
|  | 7 | 1.007(1.001, 1.013)^a^ |
| SO_2_ (lag 04) | 2 | 1.052(1.007, 1.099)^a^ |
|  | 3 | 1.052(1.007, 1.099)^a^ |
|  | 4 | 1.054(1.009, 1.101)^a^ |
|  | 5 | 1.053(1.008, 1.100)^a^ |
|  | 6 | 1.054(1.009, 1.100)^a^ |
|  | 7 | 1.052(1.007, 1.099)^a^ |
| NO_2_ (lag 04) | 2 | 1.037(1.012, 1.062)^b^ |
|  | 3 | 1.037(1.013, 1.062)^b^ |
|  | 4 | 1.039(1.015, 1.064)^b^ |
|  | 5 | 1.037(1.013, 1.062)^b^ |
|  | 6 | 1.038(1.013, 1.063)^b^ |
|  | 7 | 1.037(1.012, 1.062)^b^ |
| O_3_ (lag 05) | 2 | 1.038(1.018, 1.059)^b^ |
|  | 3 | 1.038(1.017, 1.059)^b^ |
|  | 4 | 1.033(1.012, 1.055)^b^ |
|  | 5 | 1.033(1.012, 1.054)^b^ |
|  | 6 | 1.032(1.011, 1.054)^b^ |
|  | 7 | 1.033(1.012, 1.055)^b^ |
| CO (lag 04) | 2 | 1.037(1.006, 1.068)^a^ |
|  | 3 | 1.036(1.006, 1.067)^a^ |
|  | 4 | 1.035(1.005, 1.066)^a^ |
|  | 5 | 1.034(1.004, 1.065)^a^ |
|  | 6 | 1.034(1.004, 1.066)^a^ |
|  | 7 | 1.033(1.003, 1.064)^a^ |

^a^ *p*<0.05; ^b^ *p*<0.01

**Supplemental Table 8** Sensitivity analyses for effect estimates of air pollution on non-accidental mortality by changing degrees of freedom (dfs) to control for Relative humidity (2–7)

| Variable | dfs | The estimated RRs with 95% CIs |
| --- | --- | --- |
| PM_2.5_ (lag 04) | 2 | 1.008(1.001, 1.016)^a^ |
|  | 3 | 1.008(1.001, 1.016)^a^ |
|  | 4 | 1.009(1.001, 1.016)^a^ |
|  | 5 | 1.008(1.001, 1.016)^a^ |
|  | 6 | 1.009(1.001, 1.016)^a^ |
|  | 7 | 1.008(1.000, 1.016)^a^ |
| PM_10_ (lag 05) | 2 | 1.007(1.001, 1.013)^a^ |
|  | 3 | 1.007(1.001, 1.013)^a^ |
|  | 4 | 1.007(1.001, 1.013)^a^ |
|  | 5 | 1.007(1.001, 1.013)^a^ |
|  | 6 | 1.007(1.001, 1.013)^a^ |
|  | 7 | 1.007(1.001, 1.013)^a^ |
| SO_2_ (lag 04) | 2 | 1.054(1.009, 1.100)^a^ |
|  | 3 | 1.054(1.009, 1.100)^a^ |
|  | 4 | 1.055(1.010, 1.102)^a^ |
|  | 5 | 1.054(1.009, 1.101)^a^ |
|  | 6 | 1.055(1.010, 1.102)^a^ |
|  | 7 | 1.054(1.009, 1.101)^a^ |
| NO_2_ (lag 04) | 2 | 1.038(1.013, 1.063)^b^ |
|  | 3 | 1.038(1.013, 1.063)^b^ |
|  | 4 | 1.038(1.013, 1.063)^b^ |
|  | 5 | 1.038(1.014, 1.064)^b^ |
|  | 6 | 1.038(1.014, 1.063)^b^ |
|  | 7 | 1.038(1.014, 1.063)^b^ |
| O_3_ (lag 05) | 2 | 1.032(1.011, 1.054)^b^ |
|  | 3 | 1.032(1.011, 1.054)^b^ |
|  | 4 | 1.033(1.012, 1.054)^b^ |
|  | 5 | 1.033(1.012, 1.054)^b^ |
|  | 6 | 1.034(1.012, 1.055)^b^ |
|  | 7 | 1.034(1.013, 1.056)^b^ |
| CO (lag 04) | 2 | 1.034(1.004, 1.066)^a^ |
|  | 3 | 1.034(1.004, 1.066)^a^ |
|  | 4 | 1.035(1.004, 1.066)^a^ |
|  | 5 | 1.035(1.005, 1.066)^a^ |
|  | 6 | 1.035(1.005, 1.066)^a^ |
|  | 7 | 1.033(1.003, 1.065)^a^ |

^a^ *p*<0.05; ^b^ *p*<0.01

**Supplemental Table 9** The estimated RRs with 95% CIs for daily non-accidental deaths in emergency department visits associated with a 10 μg/m^3^ increment in air pollutant concentrations (5 μg/m^3^ in SO_2_, 0.5 mg/m^3^ in CO) in multi-pollutant models

| Multi-pollutant models | Adjust for | RR (95% CI) |
| --- | --- | --- |
| PM_2.5_ (lag 04) | - | 1.008(1.001, 1.016)^a^ |
|  | +SO_2_+O_3_ | 1.009(1.001, 1.017)^a^ |
| PM_10_ (lag 05) | - | 1.007(1.001, 1.013)^a^ |
|  | +SO_2_+O_3_ | 1.007(1.001, 1.013)^a^ |
|  | +SO_2_+CO | 1.006(0.999, 1.012) |
|  | +CO+O_3_ | 1.006(0.999, 1.012) |
|  | +SO_2_+O_3_+CO | 1.006(0.999, 1.012) |
| SO_2_ (lag 04) | - | 1.054(1.009, 1.110)^a^ |
|  | +PM_2.5_+O_3_ | 1.051(1.003, 1.101)^a^ |
|  | +PM_10_+O_3_ | 1.047(1.001, 1.096)^a^ |
|  | + PM_10_+CO | 1.046(0.998, 1.095) |
|  | +PM_10_+O_3_+CO | 1.044(0.997, 1.094) |
|  | + O_3_+CO | 1.046(0.999, 1.096) |
| O_3_ (lag 05) | - | 1.032(1.011,1.054)^b^ |
|  | +PM_2.5_+SO_2_ | 1.034(1.013, 1.056)^b^ |
|  | +PM_10_+SO_2_ | 1.035(1.013, 1.056)^b^ |
|  | +PM_10_+CO | 1.035(1.014, 1.057)^b^ |
|  | +PM_10_+SO_2_+CO | 1.036(1.014, 1.057)^b^ |
|  | +SO_2_+CO | 1.035(1.014, 1.056)^b^ |
| CO (lag 04) | - | 1.034(1.004, 1.066)^a^ |
|  | +PM_10_+SO_2_ | 1.031(0.999, 1.063) |
|  | +SO_2_+O_3_ | 1.035(1.004, 1.067)^a^ |
|  | +PM_10_+O_3_ | 1.031(1.000, 1.064) |
|  | +PM_10_+SO_2_+O_3_ | 1.031(1.000, 1.064) |

^a^*p*<0.05; ^b^*p*<0.01

**Supplemental Table 10** Sensitivity analyses for effect estimates of air pollution on non-accidental mortality by using different temperature lag structures

| Variable | Temperature lag days | The estimated RRs with 95% CIs |
| --- | --- | --- |
| PM2.5 (lag 04) | Lag 0 | 1.008(1.001, 1.016)^a^ |
|  | Lag 1 | 1.007(0.999, 1.016) |
|  | Lag 3 | 1.008(0.999, 1.016) |
|  | Lag 4 | 1.008(1.000, 1.017) |
|  | Lag 5 | 1.009(1.000, 1.018)^a^ |
|  | Lag 7 | 1.010(1.001, 1.019)^a^ |
|  | Lag 9 | 1.010(1.001, 1.020)^a^ |
|  | Lag 14 | 1.014(1.004, 1.024)^b^ |
|  | Lag 21 | 1.011(1.001, 1.021)^a^ |
|  | Lag 28 | 1.015(1.005, 1.025)^b^ |
| PM10 (lag 05) | Lag 0 | 1.007(1.001, 1.013)^a^ |
|  | Lag 1 | 1.006(0.999, 1.012) |
|  | Lag 3 | 1.004(0.998, 1.011) |
|  | Lag 4 | 1.005(0.998, 1.012) |
|  | Lag 5 | 1.006(0.999, 1.012) |
|  | Lag 7 | 1.007(1.000, 1.014)^a^ |
|  | Lag 9 | 1.007(1.000, 1.014)^a^ |
|  | Lag 14 | 1.010(1.003, 1.017)^b^ |
|  | Lag 21 | 1.008(1.001, 1.015)^a^ |
|  | Lag 28 | 1.010(1.003, 1.017)^b^ |
| SO2 (lag 04) | Lag 0 | 1.054(1.009, 1.100)^a^ |
|  | Lag 1 | 1.043(0.998, 1.090) |
|  | Lag 3 | 1.031(0.987, 1.077) |
|  | Lag 4 | 1.028(0.985, 1.074) |
|  | Lag 5 | 1.034(0.990, 1.080) |
|  | Lag 7 | 1.042(0.996, 1.089) |
|  | Lag 9 | 1.046(1.000, 1.095) |
|  | Lag 14 | 1.048(0.999, 1.098) |
|  | Lag 21 | 1.050(1.001, 1.102)^a^ |
|  | Lag 28 | 1.085(1.028, 1.145)^b^ |
| NO2 (lag 04) | Lag 0 | 1.038(1.013, 1.063)^b^ |
|  | Lag 1 | 1.033(1.008, 1.058)^a^ |
|  | Lag 3 | 1.027(1.004, 1.051)^a^ |
|  | Lag 4 | 1.027(1.004, 1.051)^a^ |
|  | Lag 5 | 1.028(1.004, 1.053)^a^ |
|  | Lag 7 | 1.028(1.002, 1.053)^a^ |
|  | Lag 9 | 1.029(1.003, 1.055)^a^ |
|  | Lag 14 | 1.034(1.008, 1.060)^a^ |
|  | Lag 21 | 1.030(1.004, 1.057)^a^ |
|  | Lag 28 | 1.038(1.012, 1.065)^b^ |
| O3 (lag 05) | Lag 0 | 1.032(1.011, 1.054)^b^ |
|  | Lag 1 | 1.024(1.002, 1.047)^a^ |
|  | Lag 3 | 1.026(1.004, 1.048)^a^ |
|  | Lag 4 | 1.037(1.016, 1.059)^b^ |
|  | Lag 5 | 1.041(1.021, 1.061)^b^ |
|  | Lag 7 | 1.049(1.030, 1.068)^b^ |
|  | Lag 9 | 1.049(1.030, 1.068)^b^ |
|  | Lag 14 | 1.048(1.029, 1.067)^b^ |
|  | Lag 21 | 1.048(1.029, 1.068)^b^ |
|  | Lag 28 | 1.048(1.029, 1.067)^b^ |
| CO (lag 04) | Lag 0 | 1.034(1.004, 1.066)^a^ |
|  | Lag 1 | 1.029(0.998, 1.061) |
|  | Lag 3 | 1.029(0.998, 1.060) |
|  | Lag 4 | 1.031(0.997, 1.066) |
|  | Lag 5 | 1.034(0.997, 1.071) |
|  | Lag 7 | 1.046(1.004, 1.090)^a^ |
|  | Lag 9 | 1.067(1.018, 1.119)^b^ |
|  | Lag 14 | 1.087(1.035, 1.142)^b^ |
|  | Lag 21 | 1.081(1.028, 1.137)^b^ |
|  | Lag 28 | 1.099(1.045, 1.155)^b^ |

^a^*p*<0.05; ^b^*p*<0.01

**Supplemental Table 11** The estimated RRs with 95% CIs for daily non-accidental deaths (excluded deaths from gene-related diseases) associated with a 10 μg/m^3^ increment in air pollutant concentrations (5 μg/m^3^ in SO_2_, 0.5 mg/m^3^ in CO)

| Air pollutants |  | The estimated RRs with 95% CIs |
| --- | --- | --- |
| PM_2.5_ (lag 04) | - | 1.008(1.001, 1.016)^a^ |
|  | Exclusion | 1.009(1.001, 1.016)^a^ |
| PM_10_ (lag 05) | - | 1.007(1.001, 1.013)^a^ |
|  | Exclusion | 1.007(1.001, 1.013)^a^ |
| SO_2_ (lag 04) | - | 1.054 (1.009, 1.100)^a^ |
|  | Exclusion | 1.054 (1.009, 1.101)^a^ |
| NO_2_ (lag 04) | - | 1.038 (1.013, 1.063)^b^ |
|  | Exclusion | 1.038 (1.013, 1.063)^b^ |
| O_3_ (lag 05) | - | 1.032 (1.011, 1.054)^b^ |
|  | Exclusion | 1.033 (1.011, 1.054)^b^ |
| CO (lag 04) | - | 1.034(1.004, 1.066)^a^ |
|  | Exclusion | 1.035(1.005, 1.066)^a^ |

^a^*p*<0.05; ^b^*p*<0.01
